# Idiopathic Thrombosis of the Portal and Contributory Veins

**Published:** 1899-06

**Authors:** Bertram M. H. Rogers

**Affiliations:** Physician and Pathologist to the Royal Hospital for Sick Children and Women, Bristol.


### IDIOPATHIC THROMBOSIS OF THE PORTAL AND CONTRIBUTORY VEINS.

BY

BERTRAM M. H. ROGERS, B.A., M.D., B.Ch. Oxon.,

*Physician and Pathologist to the Royal Hospital for Sick Children and Women, Bristol.*

---

CASES of thrombosis of the portal vein, other than those caused by some septic influence, are of such rare occurrence that the subject is hardly to be found in text-books on medicine, and in the enormous mass of medical literature that now exists only some dozen cases comparable to the one to be related are to be found.

The history and course of the case which was recently under my care at the Children's Hospital is singularly complete, and consequently interesting, for the boy was under the observation either of my colleagues or myself for eight years. It is as well, perhaps, to admit at the beginning of this paper that the condition which was found *post mortem* was not diagnosed during life, but in this I do not find myself without good companions; for not only did my colleagues not recognise the true nature of the case, but in all those that I have collected from various medical publications the state of affairs was recognised but once, and that by Sir William Jenner. Whether the line of treatment adopted would have been modified had a correct diagnosis been made is another matter. This I shall refer to later.

Herbert R. was first admitted to the Hospital in October, 1891, at that time being under the care of Dr. Edgeworth, who has kindly supplied me with the notes he made at the time. The child was then 4 years and 9 months old, and had been ill for three months with increasing size of the abdomen. He was the fourth child of eight, three of whom were dead; and he is stated to have wasted from birth (a statement one often hears, but which is difficult of belief). There was no history of paternal syphilis. Examination revealed a small quantity of free fluid in the peritoneal cavity and an enlarged

[1] Read at the April Meeting of the Bristol Medico-Chirurgical Society.

spleen, whose lower edge was three inches below the costal margin. There appeared to be no disease in any other organ. There were no enlarged lymphatic glands, and no albumin in the urine. The bowels were open rather freely, the motions being a dirty yellow colour and acid in reaction. The red corpuscles were 70 per cent., the proportion of red to white 200 to 1, and the hæmoglobin 75 per cent., though a few days after the percentage was less. In December he was discharged; but in February, 1892, was re-admitted, with a history of having vomited up some blood. Examination showed that the spleen had not altered in size, but the liver was two inches below the costal margin. There was no ascites. As no hemorrhage occurred, he was sent home again in March.

In October, 1895, he was again admitted, his parents stating that he had passed some blood by the bowel. He was sick several times soon after admission, but it was not till November 9th that he brought up five ounces of pure, bright, alkaline blood, and a few hours later six more. This reduced the hæmoglobin to 20 per cent. On the 17th he brought up twenty-four ounces of blood and passed a black motion, and on the 18th, in another attack of hæmatemesis, he vomited eight ounces. This reduced him to an extreme state of anæmia, but he recovered so far that the hæmoglobin rose as high as 60 per cent., and the red corpuscles from 1,080,000 to 5,250,000, and he went on till January 13th, 1894, when he vomited twenty ounces of mixed blood and food. This was the last attack during this stay in the Hospital, and he improved so much that he went home in May. On leaving, his spleen was observed to have reached to within half an inch of the umbilicus.

In June, 1898, he came in again for a month; but keeping quite well all the time, though rather anæmic, he went home again. In November he again was admitted, after having, it was said, vomited "two quarts of blood." Whatever amount this really represents cannot be certain; but on the following day, the 9th, he brought up altogether thirty-three ounces, reducing him to such a collapsed condition that he was only brought back to life by transfusion with saline solution and inhalations of oxygen. (I may parenthetically remark that of the number of times I have employed oxygen, I believe this is the only case in which it did good, and here it was probably assisted greatly by the transfusion.) After this attack he slowly but gradually improved, and there was no evidence of hemorrhage till December 5th, when he passed black motions for two days. This hemorrhage, whenever it took place, did not seem to affect him, as his improvement continued till the 19th, when, after a very restless night, he vomited four pints and seven ounces of blood-stained fluid in the twenty-four hours. How much of this was blood cannot be said; but after the first symptom of hæmatemesis no fluid, except ice, was allowed, so that the actual quantity of blood must have been large. The next day, the 20th, he passed some bright blood—five ounces—by the bowel, and vomited thirteen ounces of pure blood. Till January 16th he was free, but on that day he began to bring up small quantities of blood, amounting in all to ten ounces. This was almost entirely clotted. He also brought up eight ounces of clear fluid, alkaline in reaction and containing a trace of albumin, the source of which I cannot explain. The following day, for the first time, his temperature rose, reaching 101°, and he passed some black fæcal matter. On the 22nd he again vomited, this time twelve ounces of bright red clotted blood. These repeated attacks of hæmatemesis reduced him to an extreme state of collapse, and, with repeated small vomits and passing of small black motions, he died on February 4th.

During all this time the physical conditions did not vary. A small amount of fluid was always present in the abdomen, which appeared to increase just before an attack of hæmatemesis came on. The spleen reached to within half an inch of the umbilicus, but the liver apparently recovered its normal size. A trace of albumin was observed at times in his urine, and his anæmic condition gave rise to hæmic murmurs.

One peculiar fact I noticed about the boy, and that was a curious earthy smell. I do not remember ever to have noticed this in any other patient, and on my calling attention to it others who happened to be with me also observed it.

The necropsy was made by me the following day. The body was very pale, and there was only slight rigor mortis. On opening the thorax a small quantity of fluid was found in each pleural cavity. The lungs were healthy; the heart was small, the right side flaccid and empty, the left full of clot; there was no pericardial fluid. No disease of the valves was present. The abdominal cavity contained several pints of clear fluid; the intestines were in a natural position, and there were no adhesions except those to be mentioned later round the spleen. The only abnormality noticed was the greatly enlarged spleen. The upper surface of this organ was firmly adherent to the diaphragm in one place, and the rest had a dull grey colour, and looked as if its surface was covered with lymph, in reality being due to the thickening of the capsule. On moving it, it was found to be also firmly adherent to the stomach, so that the two organs were removed together. Unfortunately, I did not then recognise the condition, and cut through the veins, which were thrombosed, and only removed a short portion of the duodenum. The liver, which was slightly smaller than one would have expected for a boy of this age, was pale, but otherwise normal; the gall-bladder was very large, and full of bile. The pancreas and mesentery were found to be much overgrown with fibrous tissue, and about two feet down the small intestine a deeply-congested patch, with hard knotted veins leading to it, was found and removed. The only other pathological matter of interest found was a horseshoe kidney, the second I have found while acting as pathologist to the Children's Hospital. On examining the spleen and stomach more closely, it was found that the splenic vein was full of hard discoloured clot, and that a new set of vessels had developed, which led into the spleen at various points. Tracing the vein forwards, the clot was found to extend to the vein in the small curvature of the stomach, the coronary, while, to compensate for this obstruction to the flow of blood, large veins, which were easily seen in the recent state, coursed across the stomach from the right gastro-epiploic vein. The coronary vein was blocked as far as the duodenum, but the venous communications there seem to have been sufficiently good to have prevented any engorgement. In the small intestine this did not hold good, and a branch of the superior mesenteric, which had become involved in the clot, produced a deeply-congested patch about four inches long. Here the walls of the intestine were much thickened, and the mucous membrane of a deep chocolate colour. Round the pancreas there was a considerable overgrowth of connective tissue, and the plexus of veins known as the pancreatico-duodenal and its branches were all filled with firm discoloured clot. The portal itself, as it entered the liver, was hard and cord-like, but on cutting into the liver substance it did not appear that the clot extended to any depth, nor did I find any compensatory branches. The inferior mesenteric vein was not affected.

From the cases I have found reported elsewhere, I conclude that the symptoms in this case were not unusual, but were, in fact, those seen in this condition. Hæmatemesis of more or less severe type—melæna, enlargement of the spleen, and ascites were present in every case. Osler[1] records a case in which there were hemorrhoids and no ascites, the spleen was much enlarged and adherent to the stomach and diaphragm; the liver small, and the veins of the spleen and the rectum were thrombosed. The portal vein was represented by a fibrous cord; there was no calcification of the walls, but some at the junction of the splenic and superior mesenteric. His opinion was that the primary thrombosis was portal and not splenic. In his *Principles and Practice of Medicine*, in 1892, p. 429, he says it is rarely seen except in cases of cirrhosis. These cases are rare no doubt, but from the cases I have found I should say cirrhosis was a rare cause of thrombosis, as its absence is remarked in every case except one. In Sir William Jenner's case,[2] though the patient was admittedly a heavy drinker, no cirrhosis was found. This case is a peculiar one, from the fact that the abdomen burst. This came about in the following manner: The patient was tapped for ascites, and the puncture ulcerated, and an ulcer in the intestine, caused by thrombosis of the mesenteric vein, perforated, allowing gas to escape into the peritoneal cavity, and so causing the explosion. In this case the spleen was large. Dr. Lyons[3] mentions the case of a woman aged 45, who was tapped to the extent of 500 quarts in eight months for ascites, in whom at the necropsy a clot was found in the portal vein. He particularly remarks on the absence of cirrhosis, and mentions that the liver was small. Another one is reported by Dr. Goodhart[4] as a case of cirrhosis of the liver, probably originating in phlebitis of the portal vein. Microscopical sections of the liver were shown from a woman aged 21, who in May, 1883, vomited a large quantity of blood. Diminution of the size of the liver, with splenic enlargement, came on; but on death following, only a partial examination of

[1] *J. Anat. & Physiol.*, 1882, xvi. 208.

[2] *Lancet*, 1874, i. 1. [3] *Dublin J. M. Sc.*, 1877, lxiv. 457.

[4] *Tr. Path. Soc. Lond.*, 1889, xl. 134.

the body appears to have been made, for "the portal vein trunk was not examined." It certainly seems strange to ascribe the cirrhosis to a thrombosis which possibly was never present, and certainly was never looked for.

Dr. Moorhead [1] records the case of a man aged 57, who died of hæmatemesis, and at the *post-mortem* examination the liver was normal in size and appearance—the spleen enlarged to three times its natural size. T. B. Peacock [2] remarks on the atrophy of the liver in a case of his—a man aged 52, who died of ascites, diarrhœa, and vomiting,—and believes that the condition was due to the thrombosis. S. West [3] makes much the same remarks on a case of his—a young woman with much the same symptoms as the other cases mentioned.

I think it is evident from these facts that cirrhosis from thrombosis of the portal vein is rare. That to a large amount the liver depends on the blood of the portal vein for nutrition is, I believe, a well-known fact, and should this supply be withdrawn a shrinking of the organ takes place, with impairment of function; the liver is, in fact, starved. The causes which lead to thrombosis are very obscure. In all of the cases except one I have found no reason for the condition is given, possibly because none existed. The only one solitary cause, given in one case, is indulgence in alcohol. The disease comes on apparently from no assignable cause, and usually attacks adults. My case is the youngest patient recorded; most of the others are between thirty and fifty. The course of the disease is towards a fatal issue—no case, so far as I can discover, has been recorded of a recovery. The length of the illness varies, too, considerably. Mine again holds the record for nearly eight years, most of the others are of only a month's duration or only five weeks. The longest after mine is a case recorded by G. Balfour and Grainger Stewart [4] of a young woman who, after a history of ascites for five years, died seven weeks after coming under observation. She probably, from the state of the organs found *post mortem*, had suffered from the disease all that

[1] *Tr. Path. Soc. Lond.*, 1867, xviii. 61.
[2] *Ibid.*, 1873, xxiv. 122. [3] *Ibid.*, 1878, xxix. 107.
[4] *Edinb. M. J.*, 1869, xiv. 589.

time. I see a difficulty in my case, and that is, that the periods of illness were interrupted by months of improving health. What was the condition of the boy's veins during that time cannot be said with certainty; but it is highly probable that the thrombosis existed all that time, and that from some unexplained cause, possibly some extension of the thrombosis, certain symptoms appeared at intervals. It is a point worth noting that no fever existed in any of the cases.

The question of treatment must now be considered, for it is evident that most who saw a case such as this—one with blood being vomited by the pint—would give hæmostatic drugs. As the patient is suffering from rather too much hæmostasis, that hardly appears to be right, yet I believe most would give them. I think we might say this, that drugs which are believed to cause hæmostasis by clogging the blood at the orifice of the vessel might be given with advantage; but those that are intended to act on the vessels, such as ergotine, or on the blood itself, increasing its coagulability, such as chloride of calcium, are counter-indicated.

The blood that was passed by the bowel no doubt came from the congested patch in the intestine, as well as from the stomach. It would be rather a matter of surprise if the veins of the mesentery were not thrombosed when the splenic, into which the superior generally empties its contents, was affected. In my case the inferior was not affected, but in one recorded by Barclay and Holl [1] from the duodenum downwards there was much extravasation of blood, and part of the superior and inferior mesenteric vessels were thrombosed. In Osler's case mentioned before the hemorrhoidal veins were blocked with clot, and in Payne's [2] case the superior mesenteric, and this, according to him, was the oldest part of the clot. The diagnosis, as Osler says, is rarely made, and must always be obscure. I thought in my case that the hemorrhage probably came from an ulcer in the stomach, but I could not account for the ascites or enlargement of the spleen.

[1] *Med. Times*, 1851, n. s., ii. 36.

[2] *Tr. Path. Soc. Lond.*, 1870, xxi. 228.

In the discussion which followed the reading of Dr. Rogers's paper, Dr. F. H. Edgeworth said that the chief difficulties of the case lie in the causation of the first thrombosis, which cannot have been due simply to the chlorotic condition of the blood or to the excess of the white corpuscles, but rather to some cause which produced both this and the thrombosis. It is a little remarkable that the thrombosis began in the splenic veins, for the spleen is probably an organ where a certain amount of disintegration of blood-cells goes on, and it was shown that tissue-fibrinogens will, if injected into the blood, cause an intra-vascular coagulation limited to the portal area. It is possible then that the thrombosis in the splenic veins was due to substances produced in the spleen by excessive or abnormal disintegration of blood-cells, brought about by the same cause which produced the alteration in the constitution of the blood. The changes in the blood and lymph-glands which have been observed as a result of excision of the spleen were not present in this case, unless the excess of leucocytes can be so regarded. This is probably due to the fact that the spleen was only slowly and incompletely put out of circulation owing to the opening out of new channels.—Dr. T. Fisher stated that he had seen a case diagnosed during life by Dr. Goodhart. In that case, in addition to hæmatemesis and ascites, there was a venous hum over the spleen. Dr. Fisher asked Dr. Rogers if there was any sign of re-establishment of the circulation through the thrombosed vessels to account for the temporary improvement.—Dr. Rogers, in reply, stated he had not come across the report of Dr. Goodhart's case. There did not appear to be any attempt to compensate for the thrombosis except in the spleen, to which he had called attention.

